# Bulky Substituents Promote Triplet–Triplet Annihilation Over Triplet Excimer Formation in Naphthalene Derivatives

**DOI:** 10.1021/jacs.3c08115

**Published:** 2023-09-28

**Authors:** Axel Olesund, Shima Ghasemi, Kasper Moth-Poulsen, Bo Albinsson

**Affiliations:** † Department of Chemistry and Chemical Engineering, Chalmers University of Technology, Gothenburg 412 96, Sweden; ‡ Institute of Materials Science of Barcelona, ICMAB-CSIC, Bellaterra, Barcelona 08193, Spain; § Catalan Institution for Research and Advanced Studies ICREA, Pg. Lluís Companys 23, Barcelona 08010, Spain; ∥ Department of Chemical Engineering, Universitat Politècnica de Catalunya, EEBE, Eduard Maristany 10−14, Barcelona 08019, Spain

## Abstract

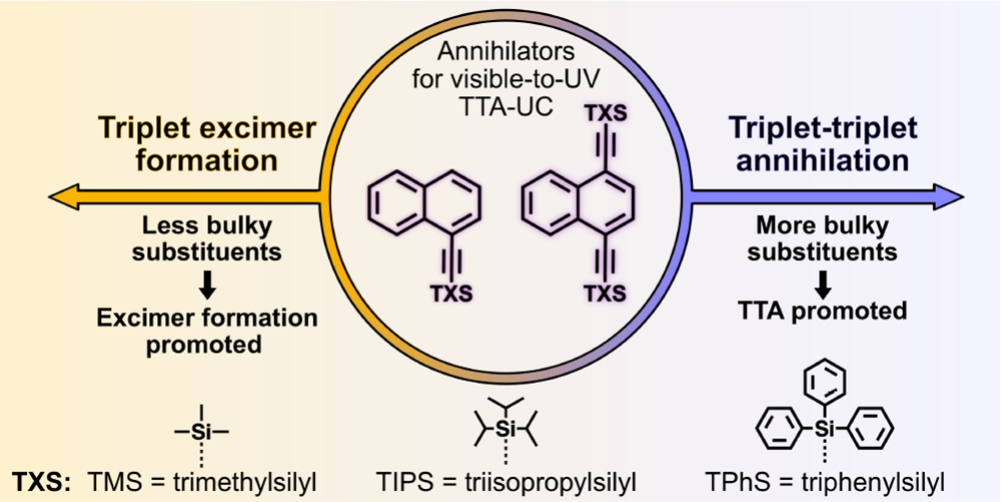

Visible-to-ultraviolet (UV) triplet–triplet annihilation photochemical upconversion (TTA-UC) has gained a lot of attention recently due to its potential for driving demanding high-energy photoreactions using low-intensity visible light. The efficiency of this process has rapidly improved in the past few years, in part thanks to the recently discovered annihilator compound 1,4-bis((triisopropylsilyl)ethynyl)naphthalene (N-2TIPS). Despite its beneficial TTA-UC characteristics, the success of N-2TIPS in this context is not yet fully understood. In this work, we seek to elucidate what role the specific type and number of substituents in naphthalene annihilator compounds play to achieve the characteristics sought after for TTA-UC. We show that the type of substituent attached to the naphthalene core is crucial for its performance as an annihilator. More specifically, we argue that the choice of substituent dictates to what degree the sensitized triplets form excimer complexes with ground state annihilators of the same type, which is a process competing with that of TTA. The addition of more bulky substituents positively impacts the upconverting ability by impeding excimer formation on the triplet surface, an effect that is enhanced with the number of substituents. The presence of triplet excimers is confirmed from transient absorption measurements, and the excimer formation rate is quantified, showing several orders of magnitude differences between different derivatives. These insights will aid in the further development of annihilator compounds for solar energy applications for which the behavior at low incident powers is of particular significance.

## Introduction

Triplet–triplet annihilation (TTA) is a process in which two dark triplet excitons interact in a spin-allowed process to form a bright and highly energetic singlet state.^1^ While it has been known to occur in many organic molecules since the 60s,^2^ research on this topic has soared in the last few decades due to its ability to partake in photochemical upconversion (TTA-UC).^3−5^ Low-energy, incident light is then transformed to light of higher energy, with potential applications in, e.g., biomedicine,^6−8^ photovoltaics,^9−12^ and photocatalysis.^7,13−17^ TTA-UC from visible to ultraviolet (UV) light is of specific interest for photocatalysis and has been proven useful for driving demanding photoreactions under mild conditions.^15,18,19^

TTA-UC requires two different species to interact with each other. The sensitizer absorbs incident light, and subsequent intersystem crossing (ISC) populates the first triplet excited state. Triplet energy transfer (TET) from the sensitizer to the annihilator species then follows, and TTA between sensitized annihilator triplets renders the high-energy emissive singlet state. To achieve an overall efficient TTA-UC process the properties of the annihilator species must fulfill certain requirements, which includes a suitable energy alignment between singlet and triplet excited states, and a high fluorescence quantum yield (Φ_F_).^20^

The efficiency of UV-emitting TTA-UC systems has improved drastically in only a few years’ time, in part due to the emergence of new materials.^18,21−24^ Naphthalene is well-known to undergo TTA^25,26^ but has rendered little success in the context of TTA-UC. Derivatives thereof have been used to some extent, with reports of TTA-UC quantum yields (Φ_UC_, here defined with a 50% theoretical maximum) ranging from below 1% (refs (19,27,28)) to well above 10% (refs (21,23,24,29)). These differences are to some degree explained by differences between the sensitizers of choice and substitution-induced differences in Φ_F_, but little to no effort has been made to understand why the TTA-UC performance of naphthalene derivatives is so drastically affected by the choice of substituents. In particular, the recent success of 1,4-bis((triisopropylsilyl)ethynyl)naphthalene (N-2TIPS) and the 3-fold inferior performance of its monosubstituted counterpart 1-((triisopropylsilyl)ethynyl)naphthalene (N-1TIPS) raises the question as to what factors affect the characteristics important for TTA-UC.^24^

**Figure 1:**
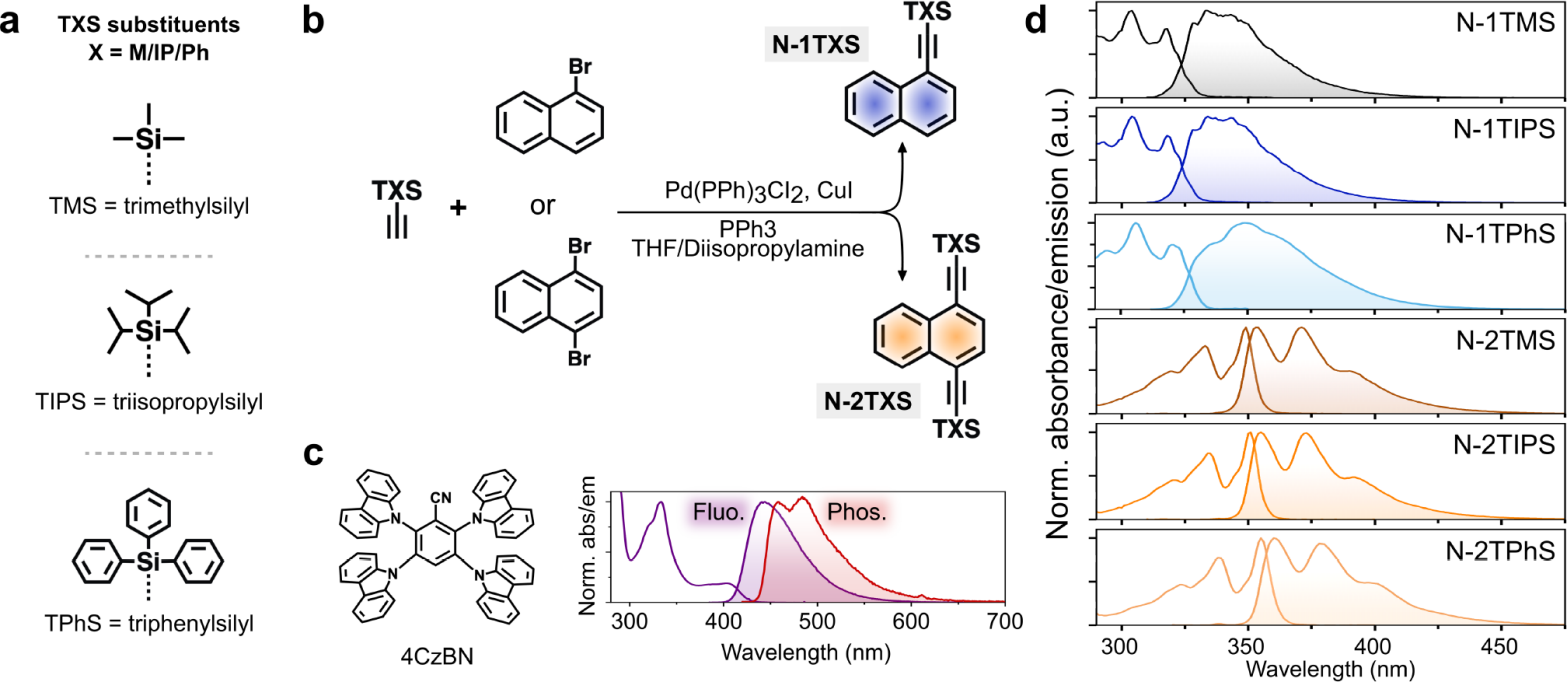
(a) Molecular structures of the TXS substituents used for the syntheses of N-1TXS and N-2TXS. (b) Simplified synthesis scheme and molecular structures of N-1TXS and N-2TXS compounds. (c) Molecular structure of the sensitizer 4CzBN and normalized steady-state absorption and emission (filled in) spectra of 4CzBN. Purple spectrum is fluorescence in toluene, and red spectrum is phosphorescence in methyl tetrahydrofuran at 100 K. (d) Normalized steady-state absorption and emission (filled in) spectra of optically dilute samples of N-1TXS and N-2TXS in toluene.

In this study, six different naphthalene derivatives with trimethylsilyl (TMS), triisopropylsilyl (TIPS), or triphenylsilyl (TPhS)-ended acetylene groups are investigated as annihilating compounds in visible-to-UV TTA-UC (Figure 1a). The derivatives are categorized in monosubstituted naphthalenes, N-1TXS (where X stands for M (methyl), IP (isopropyl), or Ph (phenyl) depending on substituent), and disubstituted naphthalenes, N-2TXS. We show that the type and number of substituents drastically affect TTA-UC performance in these compounds. An order of magnitude difference in Φ_UC_ is found between the best-performing annihilator (N-2TIPS, 16.4%) and the worst-performing one (N-1TMS, 1.3%), which is also reflected in vastly different triplet excited state lifetimes. We find that these differences emanate from the presence of a competing process, namely, excited dimer (excimer) formation between ground state and triplet excited annihilators, and that excimer formation is hampered for molecules with more bulky substituents. Triplet excimers in some TTA-UC systems have been suggested but not verified previously,^30,31^ but are herein spectrally observed by means of nanosecond transient absorption. These findings are important for the continued search for high-performing annihilator compounds, as many chromophores for TTA-UC are based on anthracene,^32−37^ naphthalene,^23,24,27,38^ or benzene,^15^ all of which are known to form triplet excimers.^39−45^

## Results and Discussion

A simplistic representation of the synthesis of the annihilators investigated is shown in Figure 1b. Mono- (N-1TXS) and disubstituted (N-2TXS) naphthalene derivatives were synthesized from Sonogashira coupling of brominated naphthalenes with different TXS substituents. Full synthesis details are found in the Supporting Information.

### Substituent Effects on the Photophysical Properties of Naphthalene Derivatives

Molecular structures of the annihilator compounds are listed in Figure 1b. If otherwise not stated, all measurements are performed in toluene, and the normalized steady-state absorption and fluorescence spectra of the annihilators are presented in Figure 1d. The choice of substituent has little to no effect on the shape and position of the electronic transitions – instead the number of substituents is what most strongly affects the shape and position of the spectra. This is expected given the characteristic behavior of the parent chromophore naphthalene, in which the S_0_–S_1_ transition is pairing forbidden in accordance with the perimeter model.^46−48^

Naphthalene has two close-lying lowest singlet electronic transitions, historically called the L_b_ and L_a_ transitions. The L_b_ transition, the lowest transition in naphthalene, is pairing forbidden and therefore has a very low molar absorptivity.^49^ This is also reflected in the long singlet lifetime and quite low fluorescence quantum yield.^50,51^ Other alternant aromatic hydrocarbons, such as benzene and pyrene, show a similar behavior stemming from the pairing symmetry of their molecular orbitals. Upon substitution at the 1 or 1,4-positions the L_a_ transition is stabilized and becomes the lowest electronic transition in the here studied substituted naphthalenes, leading to stronger absorption, shorter singlet excited state lifetimes, and higher fluorescence quantum yields. TDDFT calculations (Table S1) verify this trend, and the attractive emitter properties of, in particular, the 1,4-substituted derivatives (i.e., N-2TXS) could be ascribed to this switch in the lowest (emitting) electronic singlet state. The number of substituents seems additive, and the enhancement of absorption and emission properties is stronger in N-2TXS derivatives compared to that in N-1TXS (Table 1).

**Table 1:** Photophysical and TTA-UC Characteristics of N-1TXS and N-2TXS Derivatives and 4CzBN

	***E* (S**_**1**_**)**^a^**(eV)**	***E* (T_1_) (eV)**	***I***_**em,max**_**(nm)**	**Φ**_**F**_^c^	***k***_**TET**_^d^**(×10**^**9**^ M^–1^ s^–1^**)**	**Φ**_**UC**_^e^
N-1TMS	3.83	2.39^b^	333	0.50	2.0	0.013
N-1TIPS	3.83	2.40^f^	334	0.52	1.8	0.043
N-1TPhS	3.79	2.38^b^	349	0.50	1.6	0.050
N-2TMS	3.53	2.12^b^	353	0.77	1.1	0.078
N-2TIPS	3.52	2.12^f^	355	0.78	0.8	0.164
N-2TPhS	3.47	2.12^b^	361	0.71	1.0	0.148
4CzBN	2.99	2.71	440	0.11^g^/0.53^h^		

a First singlet excited state energy as determined from the intersection of normalized absorption and emission spectra.

b First triplet excited state energy as determined from the short-wavelength peak position of phosphorescence spectra measured in methyl tetrahydrofuran at 100 K (Figure S4a,c).

c Fluorescence quantum yield upon 307 nm excitation. Determined relative to 2-phenylindole in deaerated cyclohexane (Φ_F_ = 0.86).^51^

d Rate constant for triplet energy transfer from 4CzBN.

e Upconversion quantum yield (out of a 0.5 maximum) upon 405 nm cw excitation for samples with [S]_0_ = 25 μM and [A]_0_ = 1 mM. Determined relative to Coumarin 153 in aerated EtOH (Φ_F_ = 0.53).^53^

f ref. (24)

g Prompt component.

h Delayed component. Both components determined relative to Coumarin 153 in aerated EtOH.

**Figure 2:**
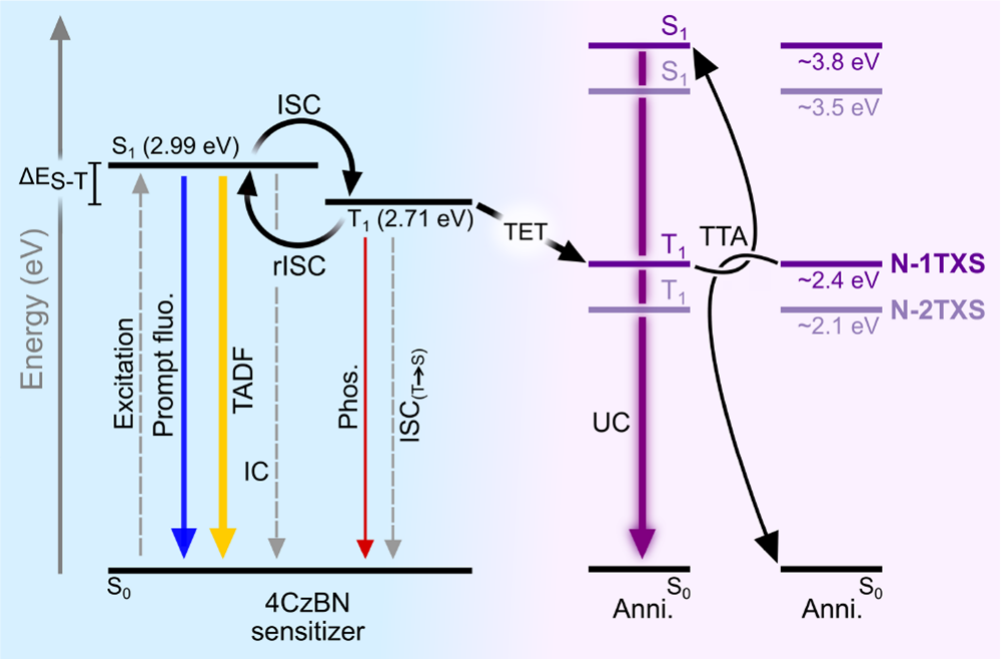
Jablonski diagram depicting the photophysical processes involved in TADF-sensitized TTA-UC. Experimentally determined singlet and triplet excited state energies are indicated for 4CzBN (black), N-1TXS (deep purple), and N-2TXS (light purple).

For sensitization, we opted for the well-known thermally activated delayed fluorescence (TADF)-type sensitizer 4CzBN (Figure 1c).^21,52^ It has previously been used with good results owing to efficient intersystem crossing (ISC, Φ_ISC_ = 0.89), a sufficiently high triplet (T_1_) energy of 2.71 eV, and an appropriately long-lived triplet state (τ_T_ = 56 μs, Figure S1). The photophysical processes involved in TADF-sensitized TTA-UC are depicted in the Jablonski diagram in Figure 2. A moderate singlet–triplet energy splitting (Δ*E*_S-T_) of 0.28 eV ensures that reverse ISC (rISC) does not outcompete TET at relevant (millimolar, mM) annihilator concentrations while keeping the energy loss during the ISC event at an acceptable level. The main purpose of using 4CzBN here is to ensure that the sensitization process is equally efficient for all annihilator species to preclude sensitizer-related differences in the overall efficiency of the TTA-UC process. Since triplet energy transfer (TET) is strongly exothermic for all annihilators (Figure 2), the small differences in measured TET rate constants (Table 1) are readily assigned only to differences in the diffusion rates of the annihilators (Figure S2). The TTA-UC relevant excited state energies of 4CzBN and the annihilators are listed in Table 1.

### Ground-State Concentration Affects the Triplet–Triplet Annihilation Upconversion Characteristics

We expected that TTA-UC would proceed with high efficiency in all annihilators, given that the thermodynamic requirement of 2 × E(T_1_) > E(S_1_) is fulfilled by a significant margin for all annihilator species. Surprisingly, this was not the case. In terms of Φ_UC_, N-2TIPS (16.4%, out of a theoretical maximum of 50%) and N-2TPhS (14.8%) far outperformed the other annihilators. The difference between N-2TIPS and N-2TPhS is readily assigned to a lower Φ_F_ value in N-2TPhS (Table 1), meaning that their capability to generate bright singlets through TTA is equally efficient. N-2TMS shows a much lower Φ_UC_ of 7.8%, despite having fluorescence that is as strong as N-2TIPS. N-1TXS annihilators perform worse generally, with Φ_UC_ at or below 5% no matter the substituent (Table 2). The presented values are that of the generated upconversion quantum yield (often referred to as Φ_UC,g_ in the literature^54^) and the fitting procedure used for obtaining these values is detailed in the Supporting Information (Figure S3). These results warrant further investigation of the kinetics involved in the TTA-UC process.

**Table 2:** Concentration-Dependent TTA-UC Characteristics of the N-1TXS and N-2TXS Derivatives

	**[A]**_**0**_^a^**(mM)**	**β**_**max**_^b^	**τ**_**T**_^c^**(ms)**	***k***_**T**_^d^**(s**^**–1**^**)**	***k***_**T0**_^e^**(s**^**–1**^**)**	***k*_exc_**^f^**(M^–1^ s^–1^)**	***k***_**TTA**_^g^**(M^–1^ s^–1^)**
N-1TMS	0.1	0.78	0.322	3.1 × 10^3^	1.9 × 10^3^	1.6 × 10^7^	4.6 ± 1.4 × 10^9^
	0.4	0.53	0.121	8.3 × 10^3^			
	0.7	0.30	0.070	1.4 × 10^4^			
	1	0.33	0.058	1.7 × 10^4^			
N-1TIPS	0.1	0.89	0.94	1.1 × 10^3^	1.03 × 10^3^	1.8 × 10^4^	5.4 ± 1.6 × 10^9^
	1	0.91	0.98	1.0 × 10^3^			
	3	0.94	0.93	1.1 × 10^3^			
	5	0.89	0.88	1.1 × 10^3^			
N-1TPhS	0.1	0.88	0.91	1.1 × 10^3^	0.95 × 10^3^	3.4 × 10^5^	5.5 ± 0.54 × 10^9^
	1	0.90	0.84	1.2 × 10^3^			
	3	0.84	0.52	1.9 × 10^3^			
	5	0.82	0.37	2.7 × 10^3^			
N-2TMS	0.1	0.90	1.50	0.67 × 10^3^	0.50 × 10^3^	1.7 × 10^6^	1.7 ± 0.24 × 10^9^
	1	0.76	0.447	2.2 × 10^3^			
	2	0.61	0.266	3.8 × 10^3^			
	3	0.51	0.178	5.6 × 10^3^			
N-2TIPS	0.1	0.95	3.60	0.29 × 10^3^	0.22 × 10^3^	5.8 × 10^4^	0.73 ± 0.10 × 10^9^
	1	0.96	3.78	0.26 × 10^3^			
	4	0.94	2.56	0.39 × 10^3^			
	8	0.89	1.39	0.63 × 10^3^			
N-2TPhS	0.1	0.93	3.72	0.27 × 10^3^	0.30 × 10^3^	4.0 × 10^4^	0.84 ± 0.11 × 10^9^
	1	0.93	2.99	0.33 × 10^3^			
	4	0.90	1.84	0.54 × 10^3^			
	8	0.89	1.69	0.63 × 10^3^			

a Ground-state annihilator concentration.

b TTA efficiency at maximum excitation power density (17.5 W cm^–2^).

c Lifetime of the first triplet excited state (T_1_).

d Pseudo-first order rate constant for the deactivation of T_1_.

e Estimated value of the rate constant of the intrinsic nonradiative decay of T_1_.

f Estimated value of the rate constant of excimer formation between T_1_ and ground state annihilators.

g Rate constant for triplet–triplet annihilation between triplet excited annihilators.

The triplet excited state lifetime (τ_T_) of the annihilator is known to be critical to the TTA-UC performance. Using a newly developed methodology, we used the time-resolved upconverted emission as a probe to determine τ_T_ of the annihilators.^55^ At 1 mM, which was the ground state annihilator concentration typically employed for TTA-UC, the annihilators exhibited vastly different τ_T_ values ranging from above 3 ms (N-2TIPS, N-2TPhS) to below 100 μs (N-1TMS, Table 2). These results roughly follow the expected trend of longer τ_T_ for the more efficient annihilators, although N-2TMS showed a factor of 2 shorter τ_T_ than N-1TIPS, despite exhibiting a significantly higher Φ_UC_. Based on molecular structure, we did not expect any big differences in τ_T_ between annihilators, and measurements of the phosphorescence decay in a frozen glass of methyl tetrahydrofuran at 100 K give lifetimes close to or in the hundreds of milliseconds, indicating that the intrinsic ability of triplets to decay to the ground state should be somewhat equal (Figure S4b,d).

We hypothesize that the observed differences in τ_T_ could emanate from interactions between annihilator triplets and annihilators in the ground state. Our suspicion grew when we realized that it has been known for many decades that neat naphthalene (and derivatives thereof) can form excited dimers, or excimers, on the triplet surface.^40−42,56^ This additional deactivation pathway for the annihilator triplets would affect the kinetics of upconversion and have an influence on the observed τ_T_. The analytical solution for the kinetic decay of upconverted emission intensity (*I*(*t*)) follows eq 1(1,57):
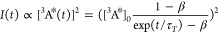
1

*I*(*t*) is quadratically dependent on the annihilator triplet concentration ([^3^A*(*t*)]), which in turn depends on τ_T_ and β, the initial fraction of annihilator triplets that undergo TTA. β can take values between 0 and 1, indicating no TTA (β = 0, triplets deactivate only through first-order processes) or deactivation only through the second-order TTA channel (β = 1). The starting concentration of annihilator triplets ([^3^A*]_0_) depends on the excitation power density (*I*_ex_) used for the measurement, and the TTA efficiency (β) is expected to increase with excitation power. We have previously shown how measurements of *I*(*t*) at different excitation powers can be globally fitted to eq 1 to yield accurate values of τ_T_.^21,33,55^

If triplet excimers are formed, an additional deactivation pathway for the triplets is introduced, and since excimer formation takes place between triplets and ground state annihilators, this process is dependent on the annihilator ground state concentration ([^1^A]_0_). Under the assumption that the formation of triplet excimers causes the individual triplets to decay, τ_T_ will be controlled by the rate of first-order triplet deactivation (*k*_T0_) as well as the pseudo first-order rate of excimer formation (*k*_exc_) according to eq 2:
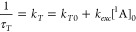
2

Equation 2 shows that τ_T_^–1^ should depend linearly on [^1^A]_0_. Determining τ_T_ at different [^1^A]_0_ values would then yield the rate of, presumed, triplet excimer formation for the different annihilators, assuming that [^1^A]_0_ is constant throughout the measurement.^39^

**Figure 3:**
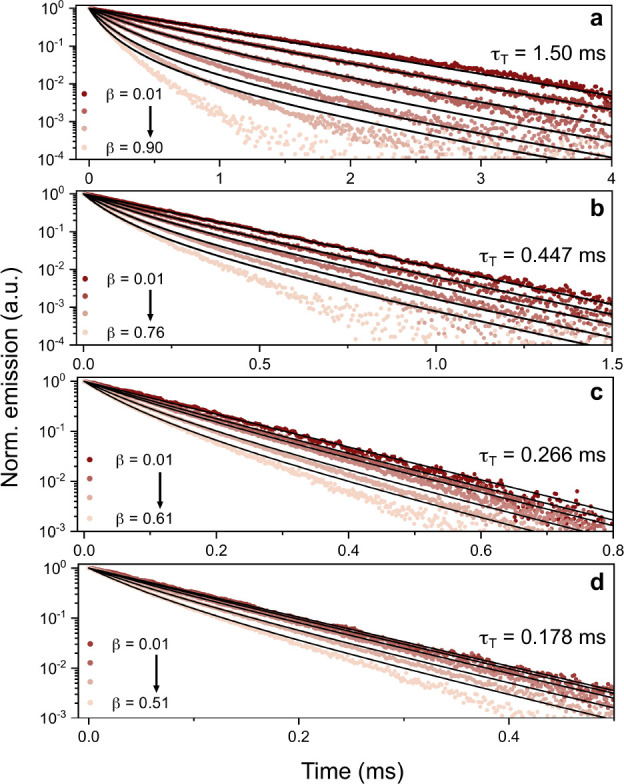
Time-resolved upconverted emission of 25 μM 4CzBN and N-2TMS for different [^1^A]_0_. (a) [^1^A]_0_ = 100 μM, (b) 1 mM, (c) 2 mM, and (d) 3 mM. Emission measured at 375 nm upon 405 nm pulsed excitation at different intensities ranging from 56 mW cm^–2^ to 17.5 W cm^–2^. Black lines are the best global fits of eq 1 using a shared τ_T_. Note that the time scales are different for each panel.

A striking example of dependence on [^1^A]_0_ is shown for N-2TMS in Figure 3. τ_T_ was initially determined to be 0.45 ms at 1 mM (Figure 3b) but is significantly increased when lowering [^1^A]_0_ to 100 μM (1.50 ms, Figure 3a) and shortened for [^1^A]_0_ at 2 and 3 mM (Figure 3c,d, respectively). The different traces correspond to different *I*_ex_ values, where higher *I*_ex_ leads to faster decays because of more efficient TTA. The larger variation in decay kinetics at low [^1^A]_0_ (Figure 3a) is expected since the influence from TTA, which in contrast to excimer formation depends on *I*_ex_, will be more pronounced (as indicated by the β values). Similar experiments were performed for all annihilators (Figures S5 to S9), showing that derivatives with TMS substituents show the strongest dependence on [^1^A]_0_. It should be noted that the global fits become increasingly worse as the emission intensity decreases and that this is expected. From a mathematical standpoint, the low-intensity data points will play an insignificant part in the global fitting procedure, whereas the fits at early times agree well with the data. Additionally, the same kind of deviation in the fittings is seen for all concentrations, indicating that the observed trends in τ_T_ are true.

**Figure 4:**
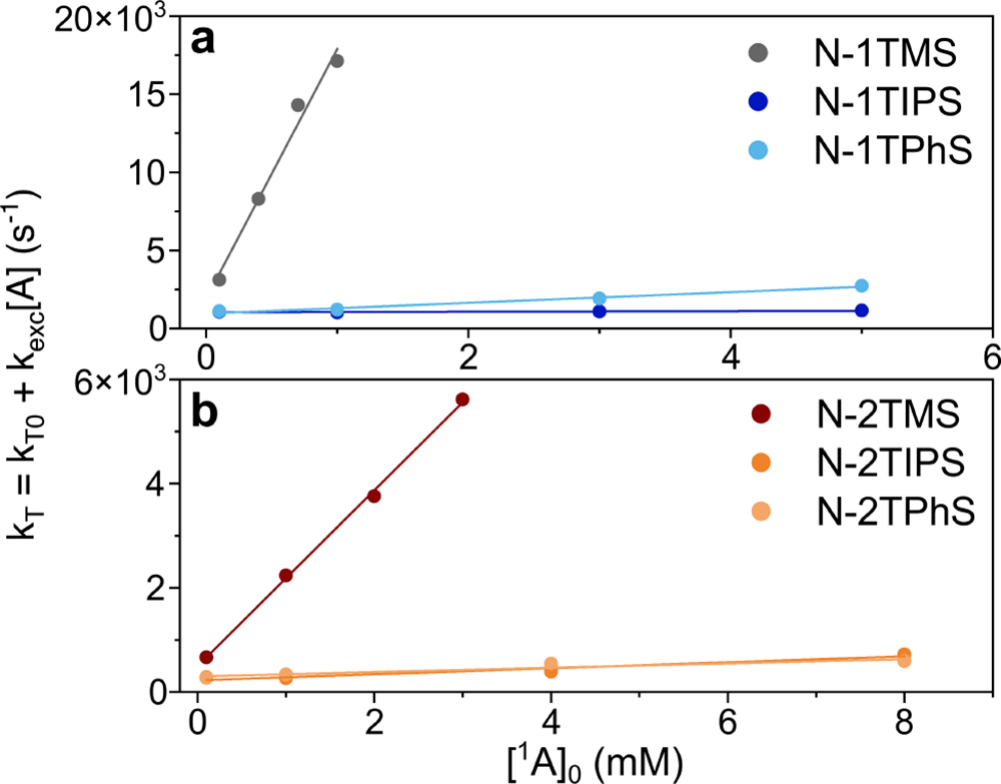
Linear fittings of eq 2 to obtained triplet lifetime data at varying [^1^A]_0_ for (a) N-1TXS and (b) N-2TXS. Note that the *y* axis scales are different for panels (a) and (b).

Figure 4 summarizes the results from the measurements of τ_T_ at different [^1^A]_0_ values. It is evident that τ_T_ is greatly affected by [^1^A]_0_ for both N-1TMS (Figure 4a) and N-2TMS (Figure 4b), resulting in a linear dependence on [^1^A]_0_ from which *k*_exc_ was extracted using eq 2. *k*_exc_ for N-1TMS and N-2TMS were 1.6 × 10^7^ and 1.7 × 10^6^ M^–1^ s^–1^, respectively, which are in the same range as the rate constant for quenching caused by ground state sensitizers.^58^ These are 2–3 orders of magnitude higher than the values obtained for the remaining annihilators. Full results from the concentration-dependent measurements are listed in Table 2. We note that the value of *k*_exc_ obtained for N-1TMS is identical to that previously determined for neat naphthalene,^41^ indicating that only one TMS substituent is insufficient to impede triplet excimer formation altogether. Virtually no difference in *k*_exc_ is seen between most TIPS- or TPhS-substituted derivatives, with N-1TPhS being the exception showing a more pronounced dependence on [^1^A]_0_ (but still much less pronounced than for both TMS-derivatives). These results indicate that it is the bulkiness of the side group that primarily affects the excimer forming ability on the triplet surface and that the number of substituents only has a minor impact. The roughly equal excimer formation ability of TIPS- and TPhS-substituted derivatives could be expected since these substituents have been shown to be equally efficient in preventing aggregation in perylene diimides (PDIs). This study also used TMS-substituents, which were not able to prevent aggregation in PDIs.^59^ Taken together, this suggests that similar substituent effects are to be expected also for annihilators based on chromophores other than naphthalene.

The rate constant for TTA (k_TTA_) was extracted from time-resolved emission measurements and the method has been explained in detail in previous publications.^21,55^ Our results indicate that k_TTA_, in contrast to *k*_exc_, primarily depends on the number of substituents but not necessarily depends on the type of substituent that is used. This indicates that TTA, like TET, is a diffusion-controlled process, as expected. The values range from 1 to 5 × 10^9^ M^–1^ s^–1^ (Table 2), which fall within the range of expected values for a diffusion-controlled process.

### Evidence of Triplet Excimer Formation in N-1TMS

The results presented so far strongly support the idea that significant self-quenching of the triplet excited state occurs in the TMS-substituted naphthalene derivatives. To verify that the self-quenching mechanism is indeed the hypothesized triplet excimer formation, we turned to nanosecond transient absorption (ns-TA). Naphthalene is well-known to show a strong triplet monomer absorption between 400 and 450 nm,^60^ whereas peaks at longer wavelengths (typically between 550 and 600 nm) have been assigned to triplet excimer absorption.^42,61−63^

**Figure 5:**
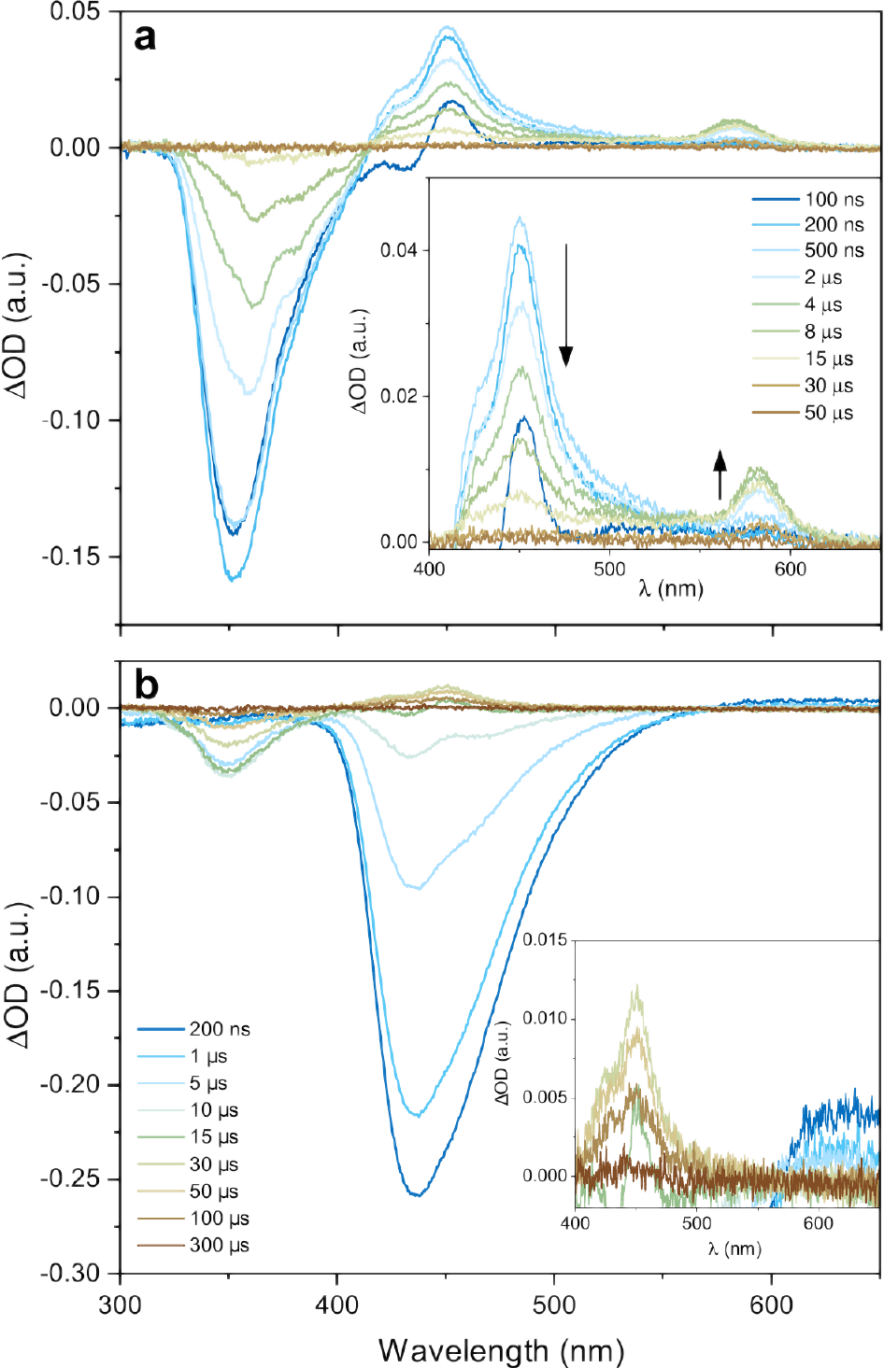
Transient absorption spectra of (a) 8 mM N-1TMS and 25 μM 4CzBN in deaerated toluene and (b) 0.1 mM N-1TMS and 25 μM 4CzBN; *l*_ex_ = 410 nm, 1.4 mJ/pulse. Insets show the absorption features of relevance.

Figure 5a shows the ns-TA spectra upon 410 nm excitation at indicated time delays for a sample consisting of 8 mM N-1TMS and 25 μM 4CzBN. Fast TET from 4CzBN triplets to N-1TMS results in expected triplet monomer absorption at 450 nm^19,61,63^ within a few hundred nanoseconds as well as upconverted emission (negative signal at 350 nm). The monomer absorption decays concomitantly with the rise of a new peak centered around 585 nm, suggesting that the 585 nm feature is formed from the triplet monomer. Very similar features have previously been observed for intramolecular dimers of dinaphthylmethane^61,63^ and naphthalenophane^62^ and have been assigned to the formation of intramolecular triplet excimers. Based on this, we assign the 585 nm peak in Figure 5a to the absorption of an intermolecular triplet excimer. The lifetime of this peak is on the microsecond time scale, suggesting that the 585 nm absorption is indeed that of a triplet state. It has previously been proposed that triplet excimers may play an important role in TTA-UC,^30,31^ but our results verify the presence of triplet excimers in a TTA-UC system for the first time.

Comparison with the spectra of a low-concentration sample (Figure 5b) further strengthens this conclusion. The spectra at early times are strongly influenced by TADF emission from 4CzBN centered at 440 nm and ground state bleach at below 380 nm, which are visible due to nonunity TET to N-1TMS. At later times, weak upconverted emission is observed at around 350 nm. However, no signs of the 585 nm absorption peak are visible at any time delay, and only the triplet monomer absorption can be observed. Since excimer formation strongly depends on ground state concentration, the absence of the 585 nm peak at low concentration further implies that the 585 nm signal is indeed the absorption of a triplet excimer.

Corresponding measurements were performed with all annihilators and are presented in Figures S10 to S14. The data suggest that none of the other derivatives form triplet excimers, including N-2TMS, which shows a strong triplet lifetime dependence on the ground state concentration (Figure 4b). Longer wavelength features are seen for all N-2TXS, but since these are (i) present already from the earliest time delays, (ii) decaying in conjunction with the 450 nm absorption feature, and (iii) present at both high and low concentration, we argue that the long-wavelength features are part of the triplet monomer absorption of N-2TXS. N-1TIPS and N-1TPhS show only a 450 nm absorption without any long-wavelength features, further suggesting that they do not form triplet excimers. It should be emphasized that triplet excimer formation is still a plausible explanation for the behavior of N-2TMS, even though this cannot be verified from the ns-TA data. No signs of singlet excimer emission, which would be expected to occur with a peak wavelength between 400 and 450 nm,^50,56,64−66^ could be detected during sensitized experiments.

The results presented above highlight the importance of avoiding triplet excimer formation to maximize the TTA-UC efficiency. However, the inferior performance of N-1TIPS cannot be explained by this process. We noted that the fluorescence decay moved from a monoexponential to a biexponential behavior when going from low (15 μM) to high (10 mM) concentration of N-1TIPS, with an additional longer-lived component appearing in the 10 mM sample (Figure S15a). The steady-state fluorescence spectra also show an enhanced intensity in the long-wavelength tail when moving to higher concentration (Figure S15b). We tentatively assign this to singlet excimer formation in N-1TIPS based on similar spectral changes being assigned to intramolecular naphthalene excimer emission previously.^67^ This could potentially disrupt the sought after TTA-UC process but warrants further investigation.

## Conclusions

In this work, we present evidence that the ability to form triplet excimers can be modulated by the choice of substituents and that the excimer formation process negatively affects TTA-UC efficiencies. In particular, six derivatives based on naphthalene have been investigated as annihilators for TTA-UC. When paired with the TADF-type sensitizer 4CzBN, TTA-UC quantum yields vary over an order of magnitude, despite all annihilators being efficiently sensitized. We show that the triplet excited state lifetime varies significantly with the annihilator ground state concentration for TMS-substituted derivatives, which we interpret in terms of enhanced formation of triplet excimers at high concentrations. We validate the presence of triplet excimers in high-concentration samples of N-1TMS by the use of nanosecond transient absorption, providing the first direct experimental evidence of triplet excimer formation in a TTA-UC system to date.

This work is envisioned to raise awareness concerning competing processes involving annihilator triplet states in TTA-UC systems. Discrepancies from expected efficiencies are often assigned to differences in the spin-statistical factor, but an in-depth understanding of observed differences is often scant. Given the propensity of naphthalene, but also benzene and anthracene, derivatives to form triplet excimers this study highlights that one must pay careful attention to, e.g., concentration effects when examining TTA-UC systems. This study also shows that triplet excimer formation is effectively turned off when bulkier substituents are added to the naphthalene core, thus providing a blueprint for the continuous improvement of annihilator designs moving forward.

## Supporting Information Available

Detailed description of experimental setups, additional spectroscopic and modeling data, calculation details, synthesis details, and proton and carbon NMR spectra (PDF)
