# Supplementary material for: Bulky Substituents Promote Triplet–Triplet Annihilation Over Triplet Excimer Formation in Naphthalene Derivatives
Source: J Am Chem Soc. 2023 Sep 28;145(40):22168–75. doi: 10.1021/jacs.3c08115 (PMC10571077; doi:10.1021/jacs.3c08115)
Supplement: Supplementary file 1 — ja3c08115_si_001.pdf [file ja3c08115_si_001.pdf]

Supporting Information

Bulky Substituents Promote Triplet-Triplet Annihilation Over Triplet Excimer Formation in Naphthalene Derivatives

Axel Olesund,[a] Shima Ghasemi,[a] Kasper Moth-Poulsen,[a,b,c,d] and Bo Albinsson[a,*]

[a]Department of Chemistry and Chemical Engineering, Chalmers University of Technology, 412 96 Gothenburg, Sweden

[b]Institute of Materials Science of Barcelona, ICMAB-CSIC, 08193 Bellaterra, Barcelona, Spain

[c]Catalan Institution for Research and Advanced Studies ICREA, Pg. Lluís Companys 23, 08010 Barcelona, Spain

[d]Department of Chemical Engineering, Universitat Politècnica de Catalunya, EEBE, Eduard Maristany 10–14, 08019 Barcelona, Spain

*E-mail: balb@chalmers.se

1. CHEMICALS, SYNTHESIS, AND EXPERIMENTAL METHODS 2

1.1 CHEMICALS 2
1.2 SYNTHESIS OF ANNIHILATORS 2
1.3 INSTRUMENTATION AND OPTICAL MEASUREMENTS 4

2. PHOTOPHYSICAL CHARACTERIZATION 5

2.1 DFT CALCULATIONS OF ANNIHILATOR EXCITED STATE ENERGIES 5
2.2 TRIPLET ENERGY TRANSFER FROM 4CZBN TO N-1TXS AND N-2TXS 6
2.3 UPCONVERSION QUANTUM YIELD DETERMINATION 7
2.4 TRIPLET STATE ENERGIES OF NEW NAPHTHALENE DERIVATIVES 9
2.5 TIME-RESOLVED UPCONVERTED EMISSION KINETICS 10
2.6 DETERMINATION OF THE K_{TTA} RATE CONSTANT 12
2.7 NANOSECOND TRANSIENT ABSORPTION (NSTA) MEASUREMENTS 13

3. ^{1}H-NMR AND ^{13}C-NMR SPECTRA 15

1. Chemicals, synthesis, and experimental methods

1.1 Chemicals

1,4-dibromonaphthalene, bis(triphenylphosphine)palladium(II) dichloride ($Pd(PPh_3)_2Cl_2$), CuI, PPh_3, diisopropylamine, triisopropylsilylacetylene, and toluene (spectroscopic grade) were purchased from Sigma-Aldrich. 2,3,5,6-tetra(9H-carbazol-9-yl)benzonitrile (4CzBN) was purchased from Ossila. Trimethyl((naphthalen-1-yl)ethynyl)silane (N-1TMS) was purchased from Sigma-Aldrich.

1.2 Synthesis of annihilators

1.2.1 General procedure

All reactions were performed under a nitrogen atmosphere using oven-dried glassware. Products were characterized by Proton (1H) and carbon (13C) nuclear magnetic resonance (NMR) using an automated Agilent Bruker (700 MHz). Dry solvents (toluene and tetrahydrofuran) were used from a MBraun MB SPS-800 solvent purification system. Column chromatography for purification was carried out using a Biotage IsoleraTM. Purification of the compounds was performed by Flash using a Biotage Isolera™ Spektra One flash chromatography system and preparative TLC plate using silica gel GF 2000 micron.

1.2.2 1,4-bis((triisopropylsilyl)ethynyl)naphthalene (N-2TIPS)

1,4-dibromonaphthalene (1.44 g, 5.03 mmol), Pd $(PPh_3)_2Cl_2$ (80.6 mg, 0.115 mmol), CuI (60.4 mg, 0.317 mmol), and PPh_3 (82.0 mg, 0.313 mmol) were dissolved in THF (40 mL) under N_2 atmosphere. After adding diisopropylamine (36 mL) to the solution, the mixture was heated to 100 °C. Subsequently, triisopropylsilylacetylene (3.52 g, 19.3 mmol) was added dropwise and the resulting solution was stirred at 100 °C overnight. After cooling to room temperature, THF and diisopropylamine were removed under reduced pressure. The residue was extracted with $CHCl_3$, dried over Na_2SO_4, filtered, and dried under reduced pressure. The crude product was purified by column chromatography (n-hexane) followed by recrystallization from methanol to give N-2TIPS (69% yield) with ≈99.9% optical purity. Three further consecutive recrystallisation steps was employed to yield N-2TIPS with an optical purity of ≈99.99% following the discovery of a fluorescent contamination. ^{1}H-NMR (CDCl3): δ (ppm) 8.41-8.38 (m, 2H), 7.63 (s, 2H), 7.61-7.58 (m, 2H), 1.20 (s, 36H), 1.19 (s, 6H). ^{13}C-NMR (CDCl3): δ (ppm) 133.01, 129.97, 127.00, 126.44, 121.60, 104.58, 97.48, 18.61, 11.24.

1.2.3 Triisopropyl(naphthalen-1-ylethynyl)silane (N-1TIPS)

1-bromonaphthalene (0.69 mL, 5.03 mmol), Pd $(PPh_3)_2Cl_2$ (80.6 mg, 0.115 mmol), CuI (60.4 mg, 0.317 mmol), and PPh_3 (82.0 mg, 0.313 mmol) were dissolved in THF (40 mL) under N_2 atmosphere. After

adding diisopropylamine (18 mL) to the solution, the mixture was heated to 100 °C. Subsequently, triisopropylsilylacetylene (2 mL ,1.70 g) was added dropwise and the resulting solution was stirred at 100 °C overnight. After cooling to room temperature, THF and diisopropylamine were removed under reduced pressure. The residue was extracted with $CHCl_3$, dried over Na_2SO_4, filtered, and dried under reduced pressure. The crude product was purified by column chromatography (n-hexane) to give N-1TIPS (75% yield). ^{1}H-NMR (CDCl3, δ): 1.21-1.27 (m, 21H), 7.45 (t, 1H), 7.55 (dd, 1H), 7.60–7.65 (m, 1H), 7.77 (d, 1H), 7.86 (t, 2H), 8.45 (d, 1H).^{13}C-NMR (100 MHz, CDCl3, δ): 11.36, 18.8, 95.7, 104.90, 121.15 , 125.07 , 126.22 , 126.27 , 126.74 , 128.18 , 128.70 , 131.95 , 133.07 , 133.44.

1.2.4 1,4-bis((trimethylsilyl)ethynyl)naphthalene (N-2TMS)

1,4-dibromonaphthalene (1.44 g, 5.03 mmol), Pd $(PPh_3)_2Cl_2$ (80.6 mg, 0.115 mmol), CuI (60.4 mg, 0.317 mmol), and PPh_3 (82.0 mg, 0.313 mmol) were dissolved in THF (40 mL) under N_2 atmosphere. After adding diisopropylamine (36 mL) to the solution, the mixture was heated to 100 °C. Subsequently, trimethylsilylacetylene (4.4 mL, 19.3 mmol) was added dropwise and the resulting solution was stirred at 100 °C overnight. After cooling to room temperature, THF and diisopropylamine were removed under reduced pressure. The residue was extracted with $CHCl_3$, dried over Na_2SO_4, filtered, and dried under reduced pressure. The crude product was purified by column chromatography (n-hexane) to give N-2TMS (69% yield). ^{1}H-NMR ($CDCl_3$, *δ*): 0.33 (s, 18 H), 7.61 (dd, 2 H), 7.62 (s, 2 H), 8.33 (dd, 2 H); ^{13}C-NMR ($CDCl_3$, *δ*):14.72, 86.42, 87.79, 106.50, 111.53, 112.24, 114.96, 118.02.

1.2.5 (Naphthalen-1-ylethynyl)triphenylsilane (N-1TPhS)

1,4-dibromonaphthalene (0.72 g, 3.5 mmol), Pd (PPh3)2Cl2 (15.2 mg, 0.08 mmol), CuI (8.4 mg, 0.12 mmol), and PPh3 (55 mg, 0.21 mmol) were dissolved in THF (20 mL) under N2 atmosphere. After adding diisopropylamine (17 mL) to the solution, the mixture was heated to 70°C. Subsequently, ethynyltriphenylsilane (1 g, 3.5 mmol) was added dropwise and the resulting solution was stirred at 70 °C overnight. After cooling to room temperature, THF and diisopropylamine were removed under reduced pressure. The residue was extracted with CHCl3, dried over Na2SO4, filtered, and dried under reduced pressure. The crude product was purified by column chromatography using Hexane/DCM (10:3). Since impurities were observed after the second column chromatography, the final product was achieved from preparative TLC using Hexane/DCM (10:3) to give N-1TPhS (30% yield). ^{1}H-NMR ($CDCl_3$): δ (ppm) 7.42-7.81(m, 18 H), 7.85-7.89(m, 3H), 8.44(d, 1H). ^{13}C-NMR ($CDCl_3$): δ (ppm): 94.17, 107.60, 120.28, 125.06, 126.18, 126.45, 127.03, 127.99, 128.27, 129.50, 129.93, 131.43, 133.02, 133.56, 135.60.

1.2.6 1,4-bis((triphenylsilyl)ethynyl)naphthalene (N-2TPhS)

1,4-dibromonaphthalene (1 g, 3.5 mmol), Pd $(PPh_3)_2Cl_2$ (147 mg, 0.21 mmol), CuI (39 mg, 0.21 mmol), and PPh_3 (55 mg, 0.21 mmol) were dissolved in THF (30 mL) under N_2 atmosphere. After adding diisopropylamine (25 mL) to the solution, the mixture was heated to 70 °C. Subsequently, ethynyltriphenylsilane (1.9 g, 7 mmol) was added dropwise and the resulting solution was stirred at 70 °C overnight. After cooling to room temperature, THF and diisopropylamine were removed under reduced pressure. The residue was extracted with $CHCl_3$, dried over Na_2SO_4, filtered, and dried under reduced pressure. The crude product was purified by column chromatography using Hexane/DCM (10:3) followed by recrystallization from (Toluene/hexane) to give N-2TPhS (40% yield). ^{1}H-NMR ($CDCl_3$): δ (ppm) 7.42-7.49(m,18 H), 7.60-7.61(m, 2H), 7.78-7.79(m, 14H), 8.44-8.45(m, 2H). ^{13}C-NMR ($CDCl_3$): δ (ppm): 96.53, 107.08, 121.61, 126.61, 127.82, 128.04, 130.02, 130.49, 133.21, 133.31. 135.60.

1.3 Instrumentation and optical measurements

Steady state absorption spectra were recorded on a Varian-Cary 50 Bio UV−vis spectrophotometer. Steady state fluorescence and phosphorescence as well as time-resolved phosphorescence measurements were carried out on a Spex Fluorolog 3 spectrofluorometer (Horiba Jobin Yvon). The prompt fluorescence lifetimes of annihilator species were measured using a spectrofluorometer (FLS1000, Edinburgh Instruments) with a 320 nm picosecond pulsed diode (Edinburgh Instruments) used for excitation.

Steady state upconversion fluorescence measurements were performed on a home-built system, consisting of a continuous-wave 405 nm OBIS laser (Coherent) as the excitation source, a 1681 SPEX monochromator, and a 9-stage photomultiplier tube (PMT) detector. The measured maximum power laser output was 88.2 mW and the laser beam diameter was 0.8 mm. In addition, to vary the laser power a linear variable neutral density (ND) filter was used, and data were recorded using home-built LabView software. A 405 nm notch filter was used in front of the detector to remove scattered laser light.

Micro- to millisecond time-resolved emission measurements were performed on the same home-built system through time-modulation of the OBIS laser using a pulse generator. The transient signals were collected with a 9-stage PMT and digitized with a 200 MHz, Tektronix MSO22 oscilloscope. The optical response time of the system was much shorter than the measured decays.

Nanosecond transient absorption measurements were performed on a home-built system using a Nd:YAG laser (Spectra-Physics, Quanta-Ray) equipped with an OPO (Spectra-Physics, primoScan) generating a 10 ns pump beam. A quartz-halogen lamp was used as the probe light. The time-resolved decays were measured on a 5-stage PMT (Applied Photophysics) coupled with a

monochromator (Oriel Cornerstone 130, Newport) and recorded on an oscilloscope (TDS 2022, Tektronix). The transient spectra were recorded on a gated ICCD camera from Andor (DH320T-25F-03, Oxford Instruments) coupled to an Andor Kymera imaging spectrometer .

All photophysical measurements were carried out in toluene using 2 mm quartz cuvettes, except nsTA measurements which were performed using 4 mm quartz cuvettes with the shorter path length directed towards the excitation pulse. All samples were prepared in a nitrogen glovebox (Innovative Technologies) with ≤0.1 ppm oxygen levels and sealed with air-tight cap screws and parafilm. Temperature-dependent measurements were performed using a liquid nitrogen cryostat (Oxford Instruments) connected to a temperature controller.

Fluorescence quantum yields of the annihilators were determined using relative actinometry, utilizing 2-phenylindole in deoxygenated cyclohexane (Φ_F = 0.86)[1] as the reference compound.

2. Photophysical characterization

2.1 DFT calculations of annihilator excited state energies

The molecular structures were geometry-optimized with DFT calculations (B3LYP/6-31G(d,p)), and vertical excitation energies and oscillator strengths (f) from the optimized ground state geometry were subsequently calculated with time-dependent DFT (B3LYP-CAM/6-311(d,p)). Even if the absolute transition energies from TDDFT calculations are approximative, and the order of close-lying excited states could be wrong, the observed trend with the strong L_a transition being stabilized and becoming the lowest transition in the substituted naphthalenes is reflecting the observed spectroscopy accurately.

Table S1. Calculated lowest singlet (S_1) and triplet (T_1) vertical excitation energies (from S_0) and oscillator strengths of the singlet transitions of naphthalene and the investigated annihilators.

	$E(S_1)$/eV (f)	$E(S_2)$/eV (f)	$E(T_1)$/eV	$E(T_2)$/eV
Naphthalene	4.62 (0.001)	4.70 (0.0761)	2.54	4.08
N-1TMS	4.22 (0.339)	4.49 (0.0015)	2.23	3.59
N-1TIPS	4.20 (0.374)	4.48 (0.0016)	2.23	3.59
N-1TPhS	4.19 (0.417)	4.48 (0.0022)	2.24	3.46
N-2TMS	3.78 (0.727)	4.35 (0.0053)	1.91	3.45
N-2TIPS	3.75 (0.695)	4.35 (0.0047)	1.91	3.45
N-2TPhS	3.72 (0.965)	4.34 (0.0079)	1.91	3.44

2.2 Triplet energy transfer from 4CzBN to N-1TXS and N-2TXS

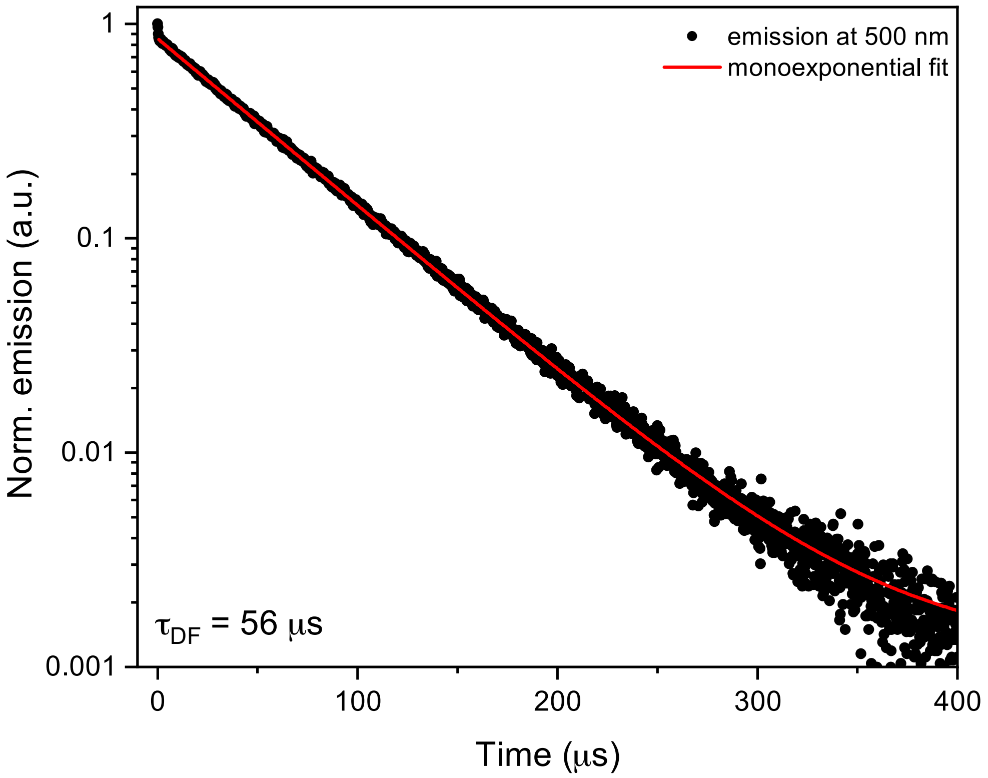

Figure S1. Time-resolved emission of 4CzBN in deaerated toluene. λ_{exc} = 405 nm, λ_{em} = 500 nm. The fast initial decay is the prompt fluorescence ($\tau_{PF} \approx 2$ ns)[2], and the fitted part of the decay is the delayed fluorescence (τ_{DF} = 56 μs).

The triplet energy transfer (TET) efficiency from 4CzBN to the annihilators was determined from quenching experiments. The addition of higher concentrations of the annihilator lead to a decrease in τ_{DF}, and the results were fitted to the Stern-Volmer equation (Eq. S1).

$$\frac{F_0}{F} = \frac{\tau_0}{\tau} = 1 + k_{TET}\tau_0[A] \tag{S1}$$

Here, F/F_0 and τ/τ_0 are the quenched/unquenched donor emission (typically phosphorescence) intensities and lifetimes, respectively, k_{TET} is the TET rate constant (M^{-1} s^{-1}), and [A] is the annihilator concentration. We used the change in τ as a proxy, as we have previously explained that this is most accurate for TADF-type donors with relatively high (>0.2 eV) singlet-triplet energy gaps.[2]

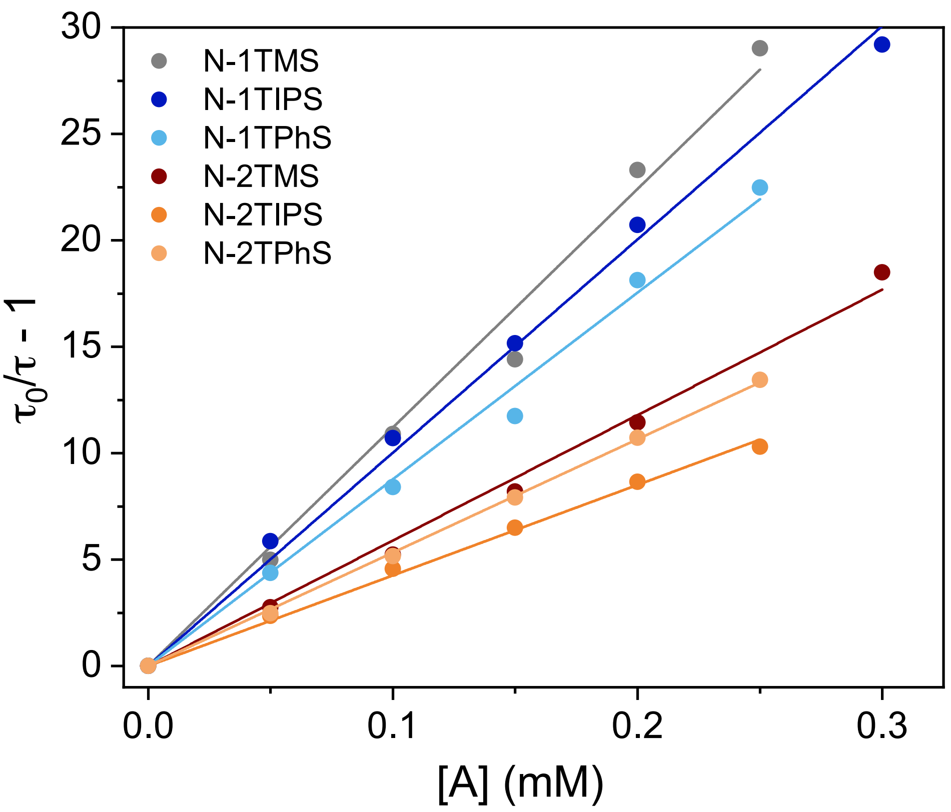

Figure S2. Stern-Volmer plots used to determine the rate of triplet energy transfer from 4CzBN to the annihilators under investigation.

2.3 Upconversion quantum yield determination

The upconversion quantum yield can be defined as in Eq. S2.

$$\Phi_{UC} = f \times \Phi_{TET} \times \Phi_{TTA} \times \Phi_F \tag{S2}$$

Here, f is the spin-statistical factor, Φ_{TET} is the TET efficiency (including ISC), Φ_{TTA} is the TTA quantum yield, and Φ_F the annihilator fluorescence quantum yield. Since two low-energy photons are needed to afford one highly energetic singlet, Φ_{TTA} (and subsequentially Φ_{UC}) has a theoretical maximum of 0.5. Many different definitions of Φ_{UC} exist, but to use Eq. S2 for analysis of, e.g., the spin-statistical factor, one must use a definition which accounts for secondary inner filter effects caused by sensitizer and annihilator reabsorption of upconverted light. This definition is often referred to as the generated UC quantum yield[3] ($\Phi_{UC,g}$), and is the value of Φ_{UC} presented in the main text. Relative actinometry was used to determine $\Phi_{UC,g}$, which was calculated using Eq. S3:

$$\Phi_{UC,g} = \Phi_r \frac{F_{UC}}{F_r} \frac{(1-10^{-A_r})}{(1-10^{-A_{UC}})} \frac{\eta_{UC}^2}{\eta_r^2} \tag{S3}$$

Here, Φ_r is the fluorescence quantum yield of the reference compound used (Coumarin 153 in air-saturated EtOH, $\Phi_r = 0.53$)[4], F_{UC} is the fitted UC emission intensities of the annihilator (blue spectra in Fig. S3), F_r is the emission intensity of the reference sample. A is the absorption of the samples at 405 nm and η is the refractive index of the solvent used, with subscripts UC and r indicating upconversion sample or reference sample, respectively.

The fitting method used to determine $\Phi_{UC,g}$ have been explained in full elsewhere.[2] Figure S3 presents measured UC spectra and the fits used to calculate Φ_{UC}. The feature peaking at 440 nm is prompt fluorescence from 4CzBN ($\Phi_F = 0.11$), which is an unavoidable loss-channel when using 4CzBN as the sensitizer.

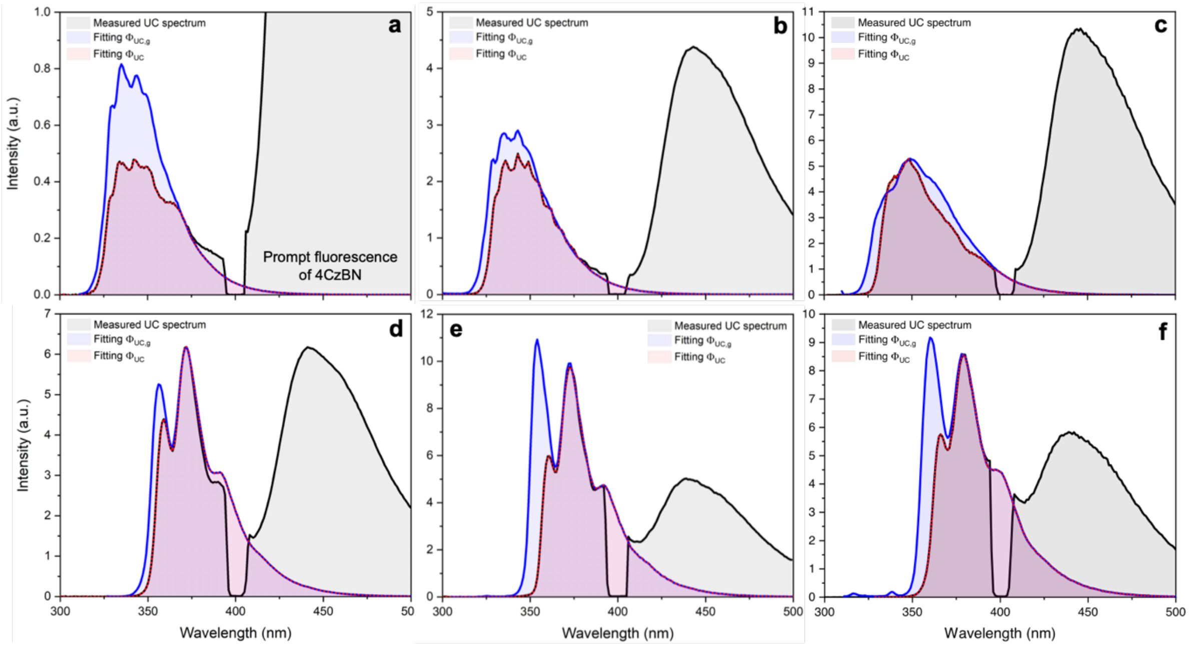

Figure S3. Upconversion spectra of 25 µM 4CzBN/1 mM annihilator in deaerated toluene. (a) N-1TMS, (b) N-1TIPS, (c) N-1TPhS, (d) N-2TMS, (e) N-2TIPS, (f) N-2TPhS, $\lambda_{exc} = 405$ nm, $I_{exc} = 17.5$ W cm^{-2}. Black spectra are from the actual measurements, red spectra are fittings to determine external UC quantum yields, and blue spectra are fittings to determine the generated UC quantum yields ($\Phi_{UC,g}$), referred to as Φ_{UC} in the main text. Φ_{UC} values are summarized in Table 1 of the main text.

2.4 Triplet state energies of new naphthalene derivatives

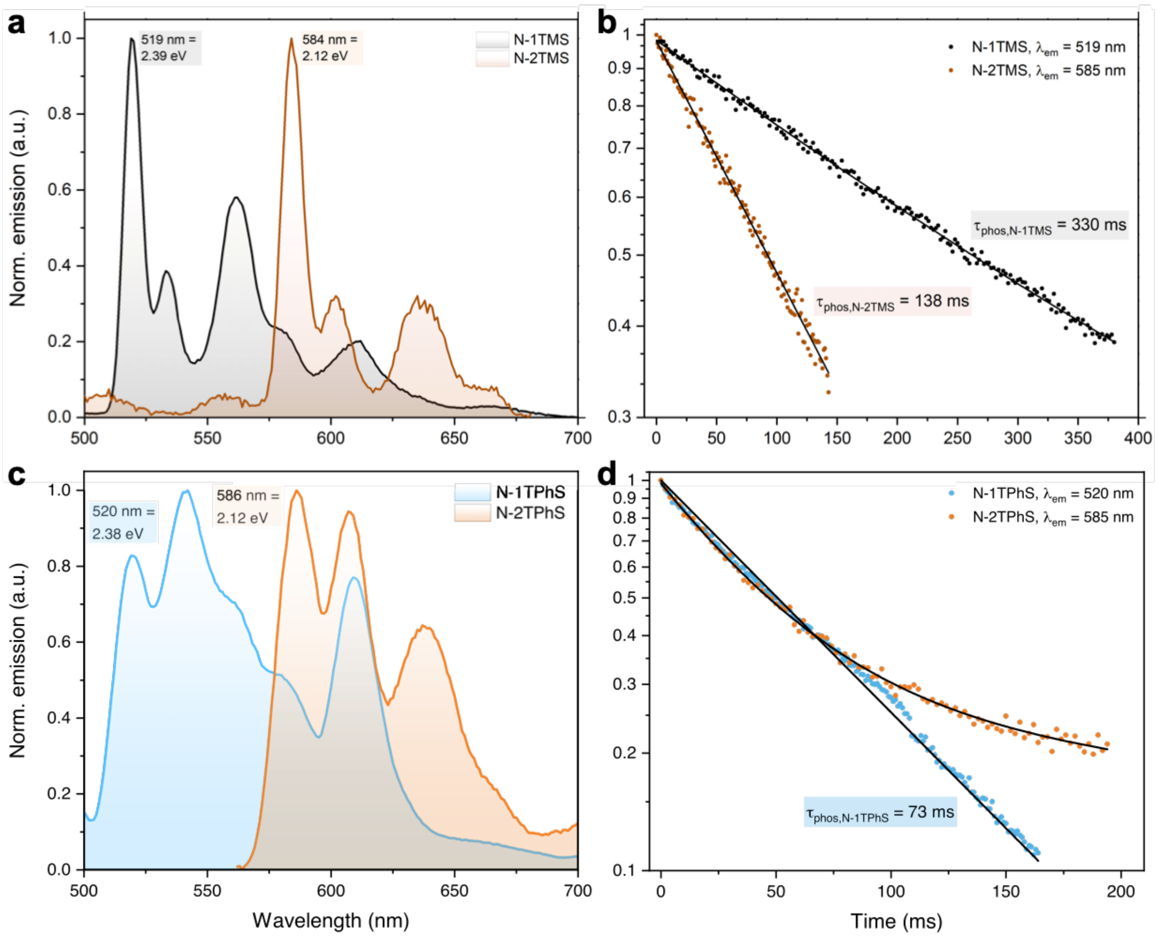

Figure S4. (a) Steady state phosphorescence spectra of N-1TMS and N-2TMS. (b) Phosphorescence decay of N-1TMS and N-2TMS. λ_{exc} = 317 nm, λ_{em} = 519 nm for N-1TMS. λ_{exc} = 332 nm, λ_{em} = 585 nm for N-2TMS. (c) Steady state phosphorescence spectra of N-1TPhS and N-2TPhS. (d) Phosphorescence decay of N-1TPhS and N-2TPhS. λ_{exc} = 319 nm, λ_{em} = 520 nm for N-1TPhS. λ_{exc} = 337 nm, λ_{em} = 585 nm for N-2TPhS. Measurements done in methyl tetrahydrofuran at 100 K.

2.5 Time-resolved upconverted emission kinetics

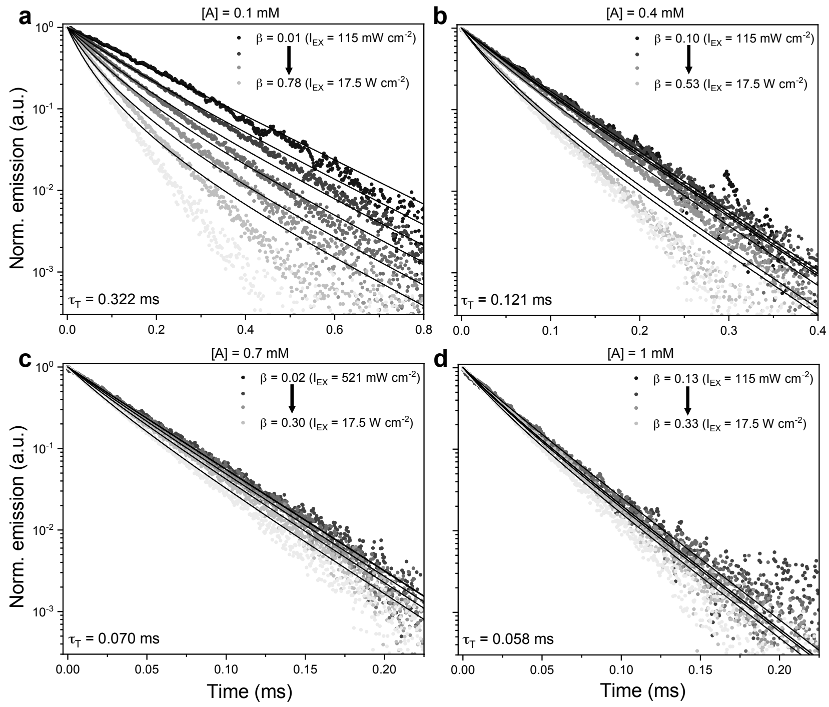

Figure S5. Upconverted emission decay kinetics of N-1TMS at different ground state concentrations using 4CzBN as sensitizer. Black lines are best global fits to Eq. 1 in the main text with a shared τ_T. λ_{exc} = 405 nm, λ_{em} = 350 nm. (a) $[A]_0$ = 0.1 mM, (b) 0.4 mM, (c) 0.7 mM, (d) 1 mM.

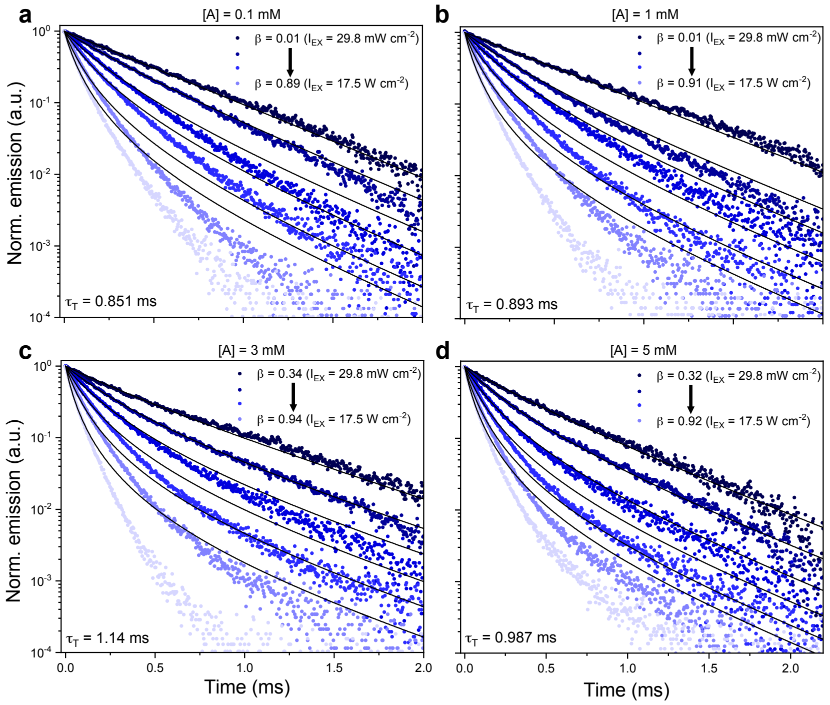

Figure S6. Upconverted emission decay kinetics of N-1TIPS at different ground state concentrations using 4CzBN as sensitizer. Black lines are best global fits to Eq. 1 in the main text with a shared τ_T. λ_{exc} = 405 nm, λ_{em} = 350 nm. (a) $[A]_0$ = 0.1 mM, (b) 1 mM, (c) 3 mM, (d) 5 mM.

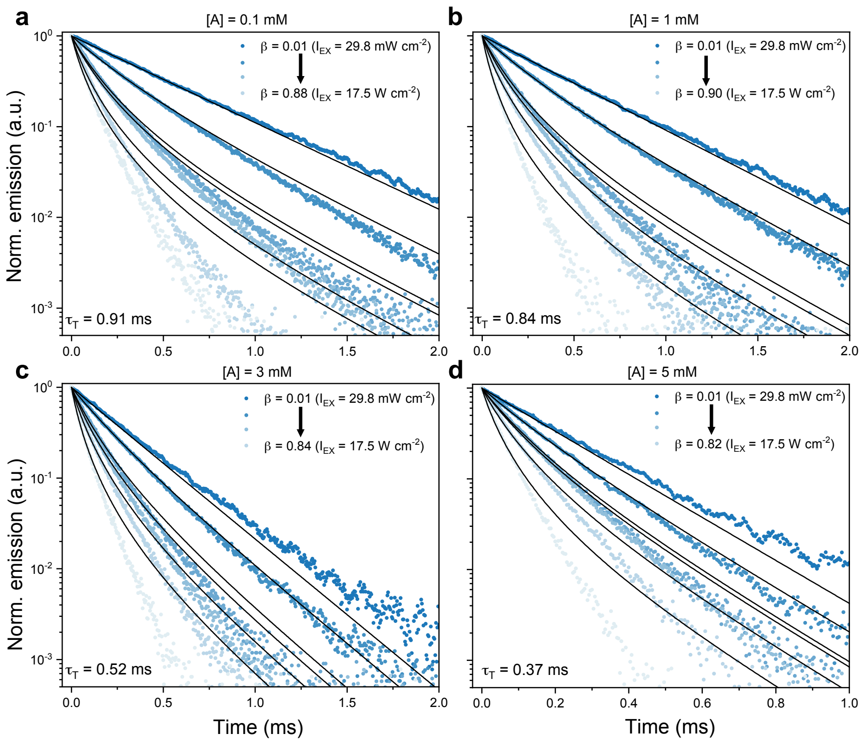

Figure S7. Upconverted emission decay kinetics of N-1TPhS at different ground state concentrations using 4CzBN as sensitizer. Black lines are best global fits to Eq. 1 in the main text with a shared τ_T. λ_{exc} = 405 nm, λ_{em} = 350 nm. (a) $[A]_0$ = 0.1 mM, (b) 1 mM, (c) 3 mM, (d) 5 mM.

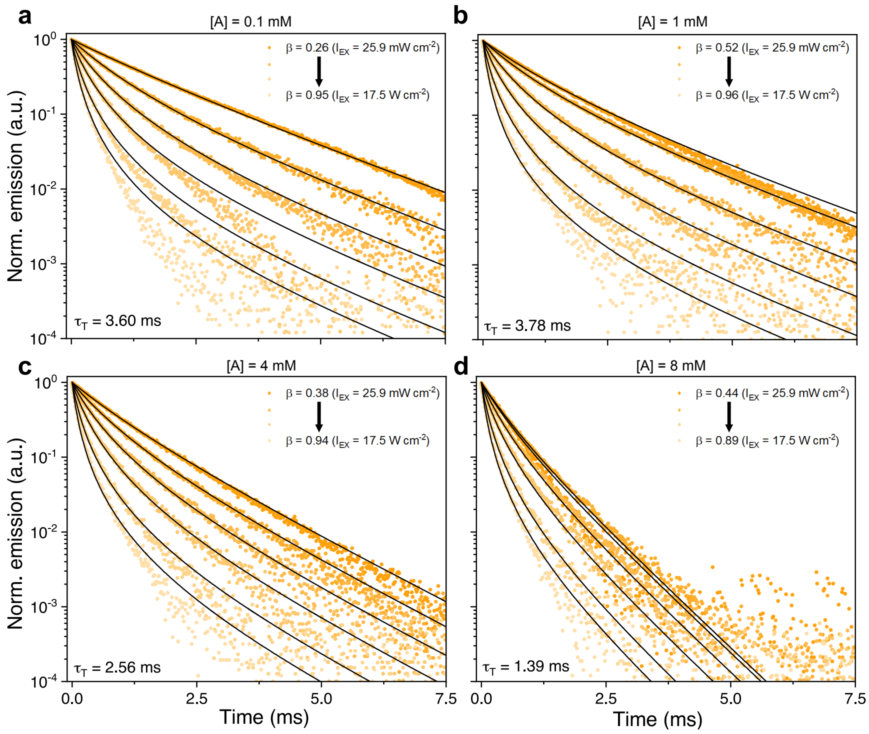

Figure S8. Upconverted emission decay kinetics of N-2TIPS at different ground state concentrations using 4CzBN as sensitizer. Black lines are best global fits to Eq. 1 in the main text with a shared τ_T. λ_{exc} = 405 nm, λ_{em} = 375 nm. (a) $[A]_0$ = 0.1 mM, (b) 1 mM, (c) 4 mM, (d) 8 mM.

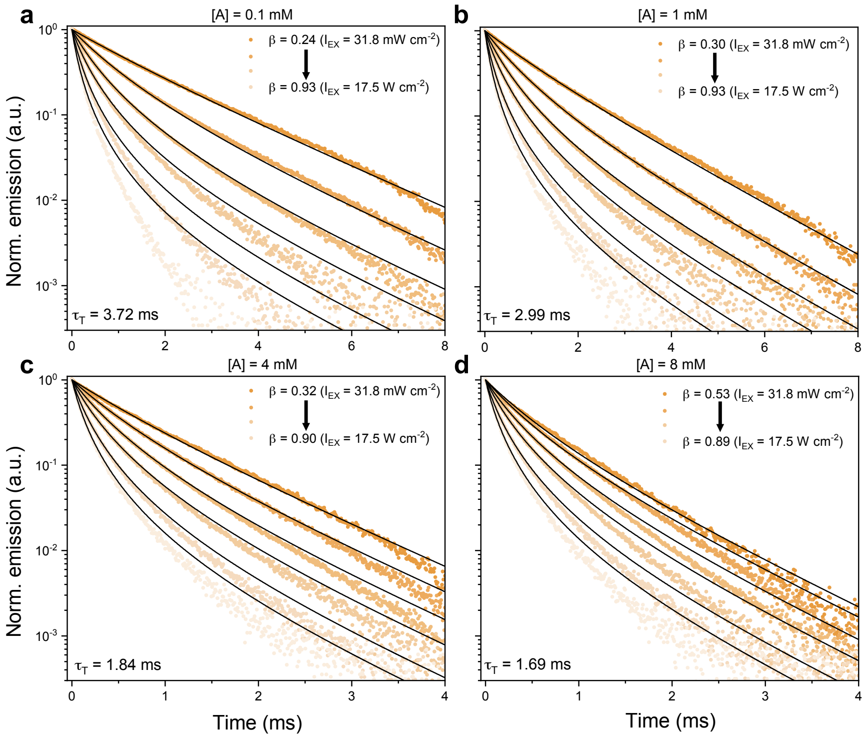

Figure S9. Upconverted emission decay kinetics of N-2TPhS at different ground state concentrations using 4CzBN as sensitizer. Black lines are best global fits to Eq. 1 in the main text with a shared τ_T. λ_{exc} = 405 nm, λ_{em} = 375 nm. (a) $[A]_0$ = 0.1 mM, (b) 1 mM, (c) 4 mM, (d) 8 mM.

2.6 Determination of the k_{TTA} rate constant

The method for determination of k_{TTA} using only time-resolved emission has been explained in full detail in a recent paper by our group,[5] and was used herein to extract k_{TTA} for all naphthalene derivatives. Briefly, by using very long square pulses (typically >1 ms) for excitation the upconverted emission intensity of the investigated system reaches a quasi steady state. By assuming that the resulting initial concentration of triplet excited state annihilators, $[^3A^*]_0$, equals that of the true steady state concentration, the collected time-resolved emission spectra (such as those in Fig. S5-S9) can be used to extract k_{TTA} by globally fitting them to Eq. 1 in the main text. Importantly, the β–value must be expressed in full in the fitting expression, according to its definition (Eq. S4).

$$\beta = 2k_{TTA}[^3A^*]_0/(2k_{TTA}[^3A^*]_0 + k_T) \tag{S4}$$

Here, k_T is the intrinsic triplet decay rate.

2.7 Nanosecond transient absorption (nsTA) measurements

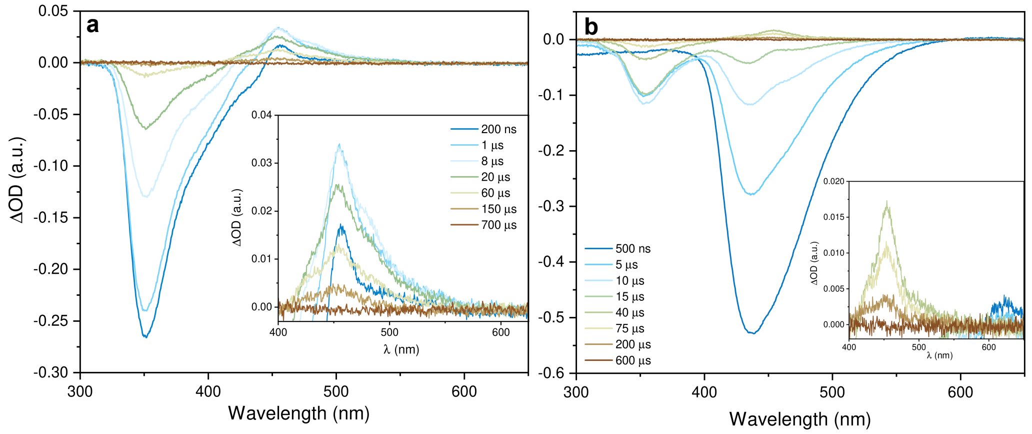

Figure S10. nsTA measurements of samples consisting of 25 µM 4CzBN and (a) 8 mM N-1TIPS, or (b) 0.1 mM N-1TIPS. λ_{exc} = 410 nm, 1.4 mJ/pulse.

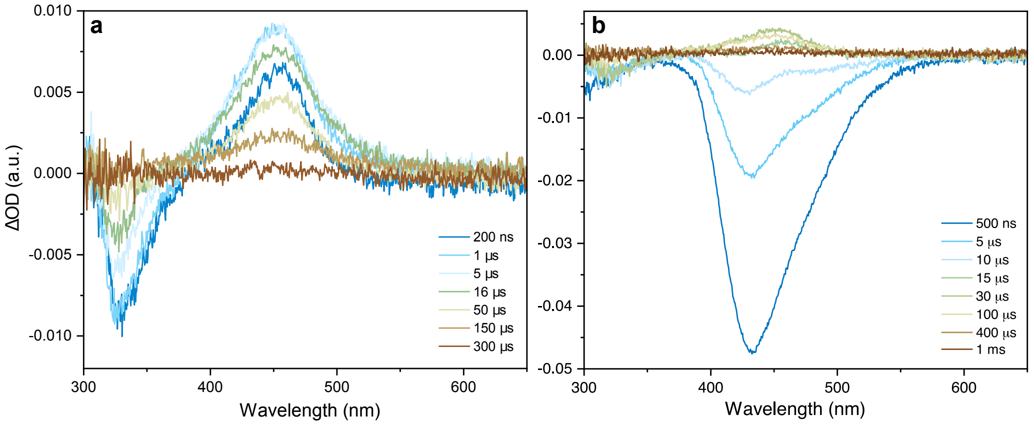

Figure S11. nsTA measurements of samples consisting of 25 µM 4CzBN and (a) 8 mM N-1TPhS, or (b) 0.1 mM N-1TPhS. λ_{exc} = 410 nm, 1.4 mJ/pulse. This measurement was performed with a Xe probe lamp, which affects the apparent emission intensities observed.

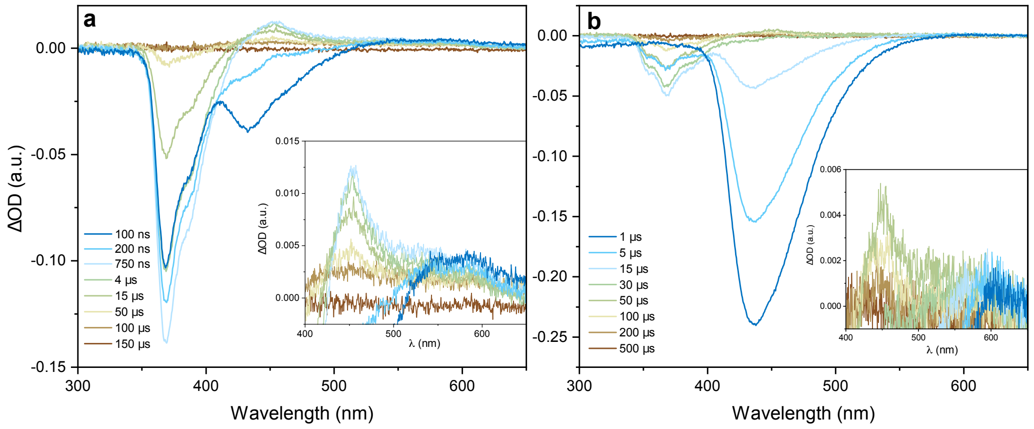

Figure S12. nsTA measurements of samples consisting of 25 µM 4CzBN and (a) 8 mM N-2TMS, or (b) 0.1 mM N-2TMS. λ_{exc} = 410 nm, 1.4 mJ/pulse.

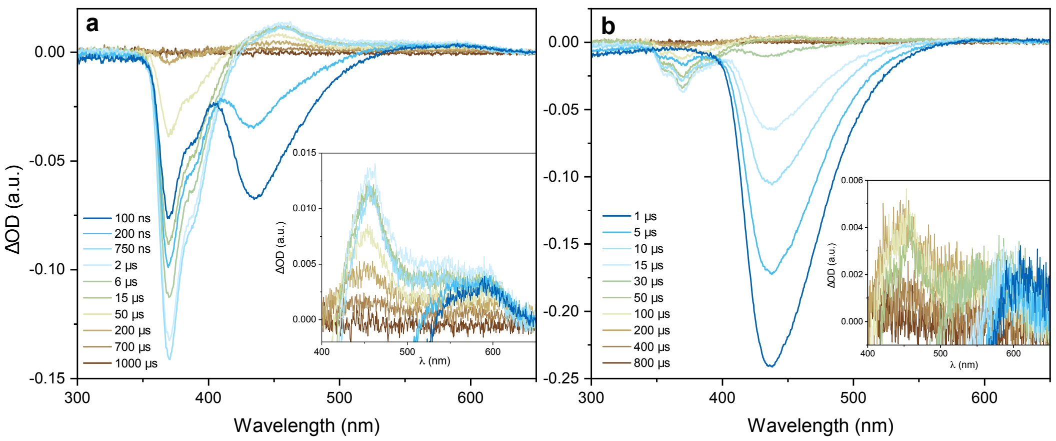

Figure S13. nsTA measurements of samples consisting of 25 µM 4CzBN and (a) 8 mM N-2TIPS, or (b) 0.1 mM N-2TIPS. λ_{exc} = 410 nm, 1.4 mJ/pulse.

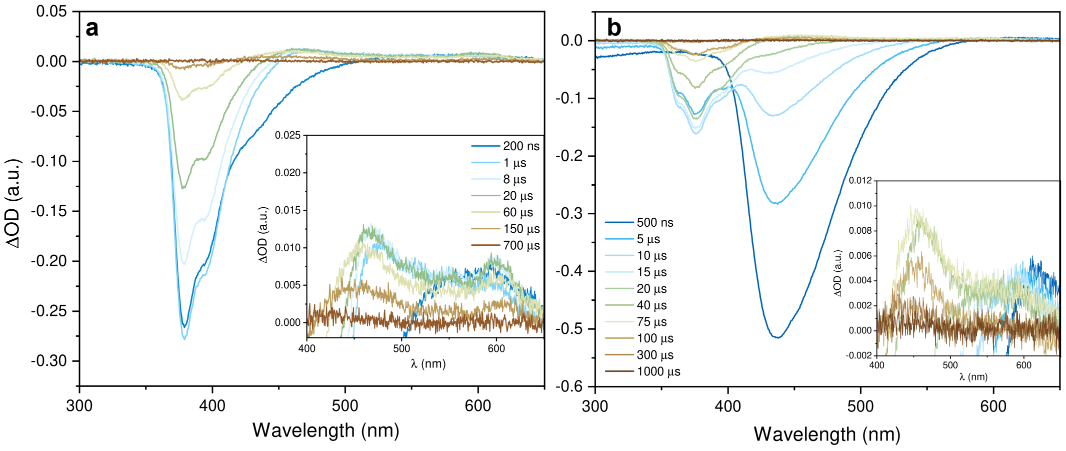

Figure S14. nsTA measurements of samples consisting of 25 µM 4CzBN and (a) 8 mM N-2TPhS, or (b) 0.1 mM N-2TPhS. λ_{exc} = 410 nm, 1.4 mJ/pulse.

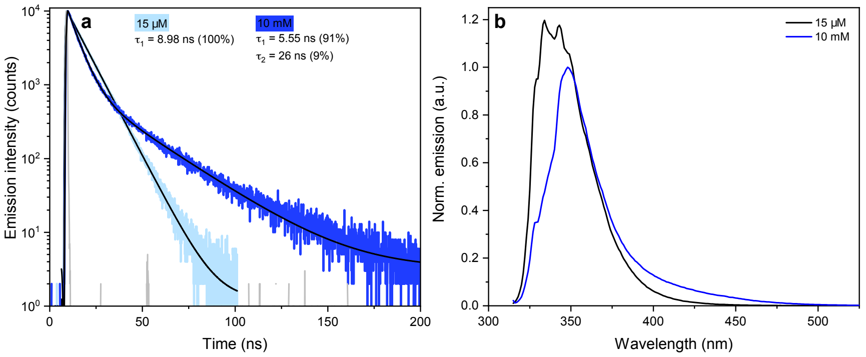

Figure S15. (a) Prompt fluorescence decay of N-1TIPS at low and high concentrations. The bi-exponential decay of the 10 mM sample is interpreted in terms of singlet excimer formation. λ_{em} = 350 nm. (b) Comparison of normalized prompt fluorescence spectra at low and high concentration of N-1TIPS. The increased intensity of the tail emission (>375 nm) is tentatively assigned to emission from a singlet excimer state. Differences in the short-wavelength (<350 nm) part of the spectra are caused by reabsorption of emitted light in the 10 mM sample. λ_{exc} = 320 nm.

3. ^{1}H-NMR and ^{13}C-NMR spectra

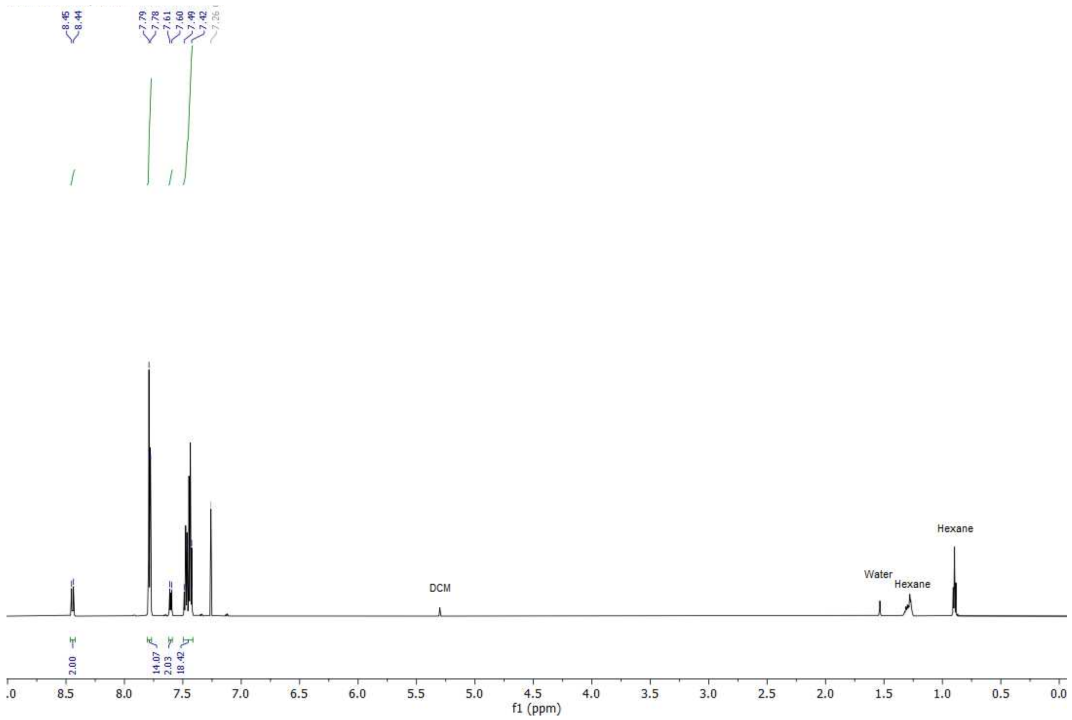

Figure S16: ^{1}H-NMR of N-2TPhS.

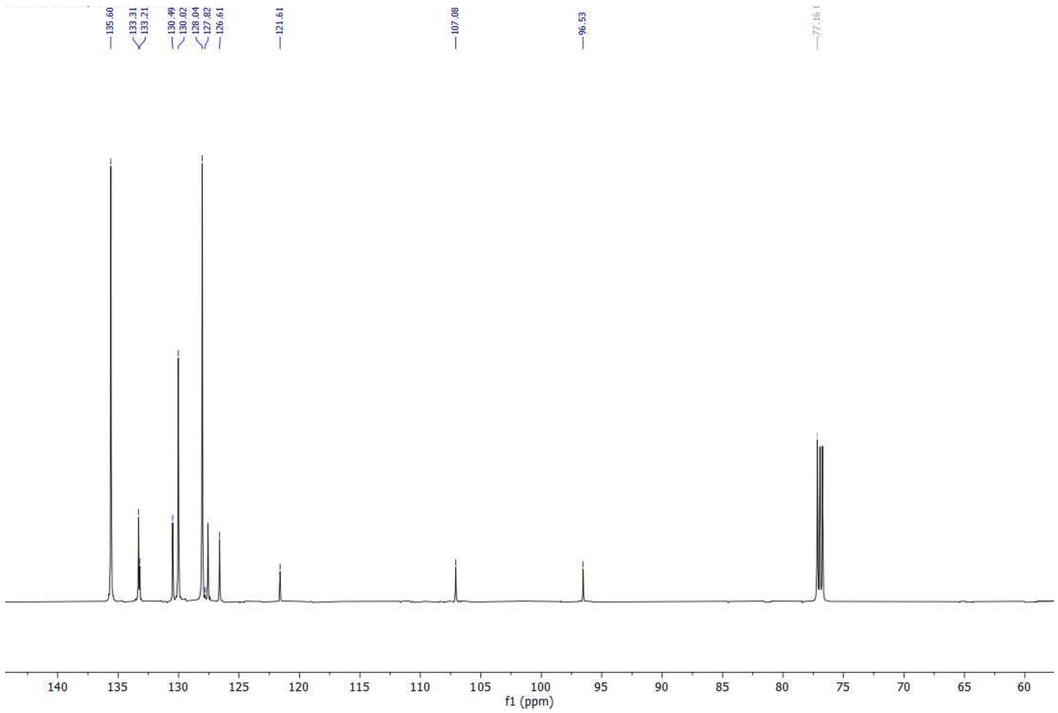

Figure S17: ^{13}C-NMR of N-2TPhS.

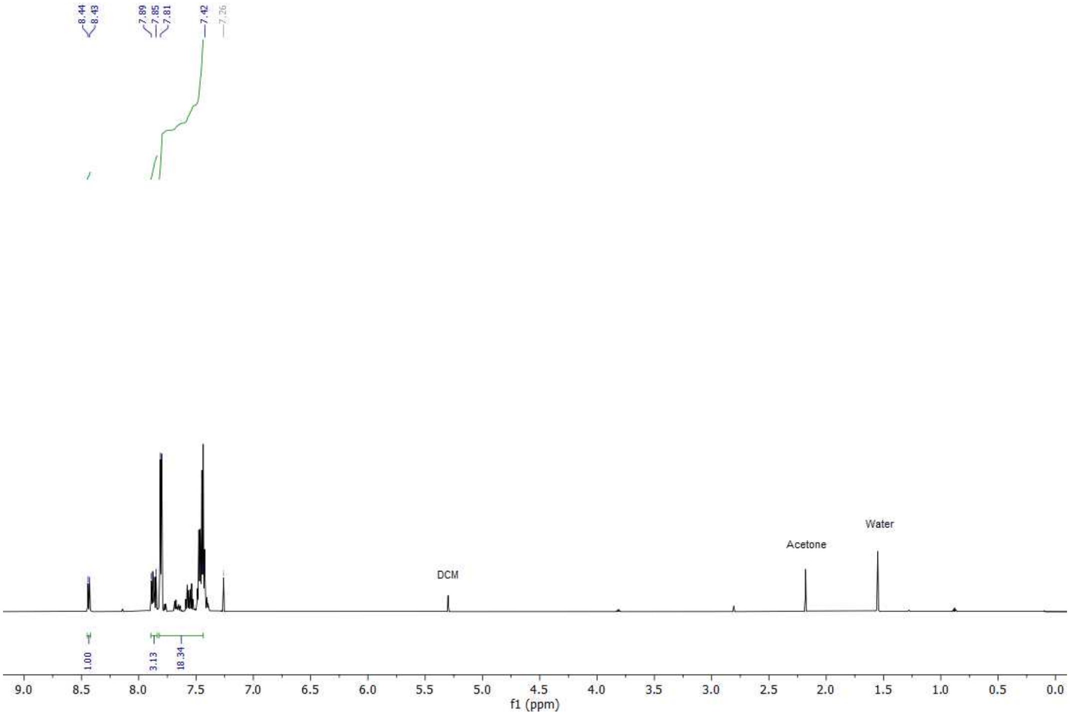

Figure S18: ^{1}H-NMR of N-1TPhS.

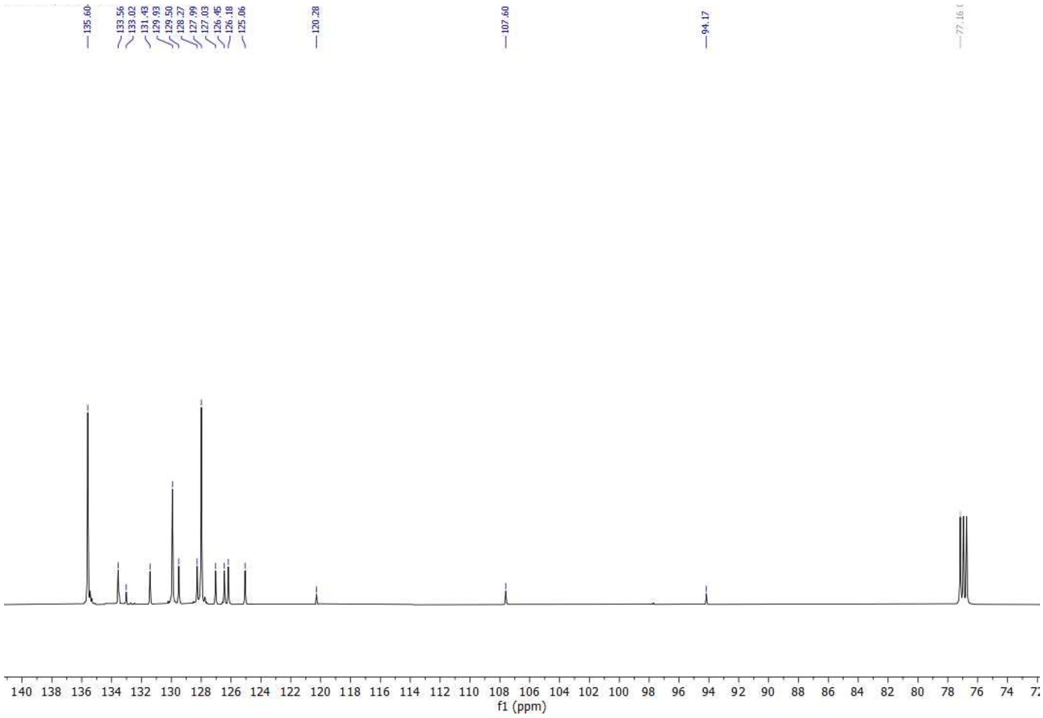

Figure S19: ^{13}C-NMR of N-1TPhS.

References

1. Berlman, I. *Handbook of Fluorescence Spectra of Aromatic Molecules*. (Academic Press: New York, 1971).
2. Olesund, A. *et al.* Approaching the Spin-Statistical Limit in Visible-to-Ultraviolet Photon Upconversion. *J. Am. Chem. Soc.* **144**, 3706–3716 (2022).
3. Zhou, Y., Castellano, F. N., Schmidt, T. W. & Hanson, K. On the Quantum Yield of Photon Upconversion via Triplet–Triplet Annihilation. *ACS Energy Letters* **5**, 2322–2326 (2020).
4. Würth, C., Grabolle, M., Pauli, J., Spieles, M. & Resch-Genger, U. Relative and absolute determination of fluorescence quantum yields of transparent samples. *Nature Protocols* **8**, 1535–1550 (2013).
5. Edhborg, F., Olesund, A. & Albinsson, B. Best practice in determining key photophysical parameters in triplet–triplet annihilation photon upconversion. *Photochem Photobiol Sci* **21**, 1143–1158 (2022).
